# Impact of the Chemokine Receptors CXCR4 and CXCR7 on Clinical Outcome in Adrenocortical Carcinoma

**DOI:** 10.3389/fendo.2020.597878

**Published:** 2020-11-13

**Authors:** Irina Chifu, Britta Heinze, Carmina T. Fuss, Katharina Lang, Matthias Kroiss, Stefan Kircher, Cristina L. Ronchi, Barbara Altieri, Andreas Schirbel, Martin Fassnacht, Stefanie Hahner

**Affiliations:** ^1^Division of Endocrinology and Diabetes, Department of Medicine I, University Hospital of Wuerzburg, University of Wuerzburg, Wuerzburg, Germany; ^2^Institute of Metabolism and Systems Research, University of Birmingham, Birmingham, United Kingdom; ^3^Centre for Endocrinology, Diabetes and Metabolism, Birmingham Health Partners, Birmingham, United Kingdom; ^4^Comprehensive Cancer Center Mainfranken, University of Wuerzburg, Wuerzburg, Germany; ^5^Institute of Pathology, Interdisciplinary Bank of Biomaterials and Data (ibdw), University of Wuerzburg, Wuerzburg, Germany; ^6^Department of Nuclear Medicine, University Hospital of Wuerzburg, University of Wuerzburg, Wuerzburg, Germany

**Keywords:** chemokine receptor, prognosis, adrenocortical carcinoma, CXCR4, CXCR7

## Abstract

Chemokine receptors have a negative impact on tumor progression in several human cancers and have therefore been of interest for molecular imaging and targeted therapy. However, their clinical and prognostic significance in adrenocortical carcinoma (ACC) is unknown. The aim of this study was to evaluate the chemokine receptor profile in ACC and to analyse its association with clinicopathological characteristics and clinical outcome. A chemokine receptor profile was initially evaluated by quantitative PCR in 4 normal adrenals, 18 ACC samples and human ACC cell line NCI-H295. High expression of CXCR4 and CXCR7 in both healthy and malignant adrenal tissue and ACC cells was confirmed. In the next step, we analyzed the expression and cellular localization of CXCR4 and CXCR7 in ACC by immunohistochemistry in 187 and 84 samples, respectively. These results were correlated with clinicopathological parameters and survival outcome. We detected strong membrane expression of CXCR4 and CXCR7 in 50% of ACC samples. Strong cytoplasmic CXCR4 staining was more frequent among samples derived from metastases compared to primaries (*p=*0.01) and local recurrences (*p=*0.04). CXCR4 membrane staining positively correlated with proliferation index Ki67 (r=0.17, *p=*0.028). CXCR7 membrane staining negatively correlated with Ki67 (r=−0.254, *p=*0.03) but positively with tumor size (r=0.3, *p=*0.02). No differences in progression-free or overall survival were observed between patients with strong and weak staining intensities for CXCR4 or CXCR7. Taken together, high expression of CXCR4 and CXCR7 in both local tumors and metastases suggests that some ACC patients might benefit from CXCR4/CXCR7-targeted therapy.

## Introduction

Chemokines and their receptors play a major role in immune cell trafficking in both physiological and pathological settings (1, 2). They are an active component of the tumor microenvironment, driving tumor-specific immune responses and promoting invasion, metastasis, stemness and resistance to chemo- and radiotherapy (1, 2). Recently, expression of CXCR4 was reported in primary tumors and metastatic lesions of patients with ACC both at protein level *in vitro* and *in vivo* using radiolabeled CXCR4 ligands (3, 4).

CXCR4, a classical transmembrane G protein-coupled receptor, has been associated with more aggressive tumor phenotypes and poor prognosis in several cancer types (5–8). Its ligand CXCL12 (SDF-1) is highly abundant in tissues that are common sites of metastasis such as lymph nodes, lung or bone, suggesting a specific chemokine-mediated trafficking-pattern of circulating tumor cells (6, 9, 10). CXCR7, an atypical chemokine receptor with a ten times higher affinity for CXCL12 compared to CXCR4, was detected at protein level in ACC metastases and correlated with CXCR4 expression (4). CXCR7 can generate CXCL12 gradients for CXCR4 but also acts as a CXCL12 “scavenger”, as it is constantly recycled to the cell membrane after ligand binding (11, 12). In cancer, CXCR7 mainly promotes local tumor growth and angiogenesis (11–13).

In recent years, CXCR4 has emerged as a potential target for cancer treatment with a particular focus on cancer stem cells that are regarded as chemotherapy**-**resistant (14–17) and several CXCR4 antagonists have shown promising therapeutic effects in first studies (18–20). Furthermore, radiotracers for non-invasive *in vivo* characterization of CXCR4 expression have entered clinical evaluation (21–25). However, only one CXCR4 antagonist (Plerixafor®) has been approved for therapeutic purposes for stem cell apheresis in multiple myeloma and lymphoma (26). The main limitations in developing a CXCR4 and/or CXCR7-targeted therapy are not only of biological nature due to the important roles of both chemokine receptors in the normal physiology, but also due to technical limitations. Only few antibodies are available and the prognostic impact of CXCR4 and CXCR7 is not consistent among different cellular localizations. Cell membrane localization mostly reflects the activated state of the chemokine receptor and has been associated with a worse prognosis in esophageal cancer for CXCR7 and in gastric and breast cancer for CXCR4 especially due to enhanced metastasis (27–29). On the contrary, high cytoplasmic CXCR4 localization was reported to be favorable for triple-negative breast cancer and adenocarcinoma of the lung (30, 31) but has been independently associated with lymph node metastasis of breast cancer in another analysis (32).

The aim of our study was to describe the chemokine receptor profile in ACC, focusing in particular on CXCR4 and CXCR7 and their prognostic relevance.

## Material and Methods

### Study Subjects

We included patients with histologically confirmed ACC and available formalin fixed paraffin-embedded (FFPE) specimens, who were treated at our center since 2004. The following clinical and histopathological characteristics were assessed: sex, age at diagnosis, tumor size, Ki67 proliferation index, Weiss score, staging according to ENSAT classification (33), hormone secretion, presence of distant metastases and specific anti-tumor treatments (**Table 1**). The study was approved by the ethics committee of the University of Wuerzburg (No. 88/11). Patients had given written informed consent for tissue collection and analysis of clinical data.

**Table 1:** Clinical parameters of ACC patients (n=187).

Sex	
Male, n (%)	62 (33)
Age at diagnosis, y (mean±SD)	49±15
ENSAT stage, n (%)	
I	12 (6)
II	85 (45)
III	40 (21)
IV	53 (28)
Unknown	5 (3)
Tumor size (cm), mean±SD	12±5.4
Hormone secretion, n (%)	
Yes	98 (52)
Cortisol	75 (77)
Androgens/estrogens/progesterone	54 (55)
Mineralocorticoids	9 (9)
No	33 (18)
Unknown	56 (30)
Ki67 (%), n (%)	
Low (<10)	38 (20)
High (≥10)	125 (67)
Unknown	24 (13)
Weiss score^a^, n (%)	
Low (≤6)	100 (53)
High (>6)	52 (28)
Unknown	35 (19)
Resection status, n (%)	
R0	94 (50)
R1	16 (9)
R2	25 (13)
Rx	20 (11)
Unknown	30 (16)
Surgically not removed	2 (1)
Mitotane, n (%)	
Yes	153 (82)
No	21 (11)
Unknown	13 (7)
Chemotherapy, n (%)	
No	48 (26)
Unknown	14 (7)
Yes	125 (67)
EDP^b^	99 (79)
EP^c^	11 (9)
Gemcitabine/Capecitabine	60 (48)
Streptozotocin	68 (54)
Other	61 (49)
Radiotherapy (primary tumor and/or metastases), n (%)	
Yes	52 (28)
No	121 (65)
Unknown	14 (7)
Additional surgery, n (%)	
Yes	71 (38)
No	103 (55)
Unknown	13 (7)

a Divided into low and high according to mean.

b E, etoposide; D, doxorubicin; P, platinum compound (Cisplatin/Carboplatin).

c E, etoposide; P, platinum compound (Cisplatin/Carboplatin).

### Gene Expression Analysis

Chemokine receptor mRNA expression levels were investigated by quantitative real-time polymerase chain reaction (qRT-PCR). Adrenocortical tissue is composed of different cell entities and leukocyte infiltration in tumor tissue might have influenced chemokine receptor levels detected by qRT-PCR. We therefore also analyzed the chemokine receptor profile in a total of 13 adrenocortical NCI-H295 cancer cell line samples obtained from 3 different sources. RNA was isolated from fresh frozen tissue of eighteen ACCs (not included in the IHC cohort) and four normal human adrenal glands using the RNeasy Lipid Tissue Minikit (Qiagen, Hilden, Germany) and from the human adrenocortical cancer cell line NCI-H295 using the RNeasy Mini Kit (Qiuagen). Reverse transcription of RNA was performed using the QuantiTect Reverse Transcription Kit (Qiagen), as previously described (34). The following Taqman Gene Expression assays from Applied Biosystems (Darmstadt, Germany) were used to analyze the chemokine receptor profile: *CCR1* (Hs 00928897_s1), *CCR2* (Hs 00704702_s1), *CCR3* (Hs 01847760_s1), *CCR4* (Hs 00747615_s1), *CCR5* (Hs99999149_s1), *CCR6* (Hs 10890706_s1), *CCR7* (Hs01013469_m1), *CCR8* (Hs 00174764_m1), *CCR9* (Hs01890924_s1), *CCR10* (Hs00706455_s1), *CCR11* (Hs00664347_s1), *CXCR1* (Hs 01921207_s1), *CXCR2* (Hs 01891184_s1), *CXCR3* (Hs01847760_s1), *CXCR4* (Hs00607978_s1), *CXCR5* (Hs00540548_s1), *CXCR6* (Hs01890898_s1), *CXCR7* (Hs00664172_s1) and *CX3CR1* (Hs 01922583_s1). Endogenously expressed *β-actin* (Hs9999903_m1) was used for normalization. 40 ng cDNA was used for each PCR reaction. qRT-PCR was performed three times for each cell line. Transcript levels were determined using the TaqMan Gene Expression Master Mix (Applied Biosystems), the CFX96 real-time thermocycler (Bio-rad, Hercules, CA, USA) and Bio-Rad CFX Manager 2.0 software. Cycling conditions were 95°C for three min followed by 50 cycles of 95°C for 30 s, 60°C for 30 s, and 72°C for 30 s. Using the ΔCT method, the gene expression levels were normalized to those of *β-actin*, as previously described (35).

### NCI-H295 Cell Culture

The human adrenocortical cancer cell line NCI-H295 was obtained from American Type Culture Collection (ATCC, Rockville, MD, USA). NCI-H295 cells were cultured with RPMI-1640 medium supplemented with 10% FCS, insulin (5 µg/ml), transferrin (100 µg/ml) and sodium selenite (5.2 ng/ml). Medium was changed every 48–72 h. 30% of the conditioned culture medium was used for passaging. Cells were frozen for RNA extraction. Short tandem repeat-profiling confirmation was performed.

### Immunohistochemistry

Immunohistochemistry was performed in 187 FFPE unmatched ACC specimens (159 primary tumors, 17 local recurrences, 11 metastases). Standard full slides (n=95) were available for the analysis of both chemokine receptors. Staining for CXCR7 was evaluable in n=84. The expression of CXCR4 was additionally assessed on tissue microarrays (TMA) (n=92). The tissue sections were deparaffinized in xylene and rehydrated in ethanol (100, 90, 80, and 70% each concentration for 5 min). Immunohistochemical detection was performed using an indirect immunoperoxidase technique after high temperature antigen retrieval in 10 mM citric acid monohydrate buffer (pH 6.5) in a pressure cooker for 13 min. Blocking of unspecific protein-antibody interactions was performed with 20% human AB serum in PBS for 1 h at room temperature (RT). Primary CXCR4 antibody (Abcam, UMB2; 124824) and CXCR7 antibody (Abcam, 38089) were used at a dilution of 1:100 at RT for 1 h. Signal amplification was achieved by En-Vision System Labeled Polymer-HRP (Dako) for 40 min and developed for 10 min with DAB Substrate Kit (Vector Laboratories, Burlingame, CA, USA) according to the manufacturer’s instructions. Mayer’s hematoxylin was used for the counterstaining of nuclei. Negative controls were carried out by treating the slides with N-Universal Negative Control Anti-Rabbit (Dako, Glostrup, Denmark) instead of the primary antibody, yielding a nearly complete loss of staining with only some faint background.

All slides were evaluated independently by three investigators blinded to patients’ clinicopathological data. Staining intensity was evaluated with a grading score of 0, 1, 2, or 3, which corresponded to negative, weak, moderate, or strong staining intensity, respectively. The percentage of positive tumor cells was calculated for each specimen and scored 0 if 0% were positive, 0.1 if 1–9%, 0.5 if 10–49%, and 1 if ≥ 50%. A semi-quantitative H-score was then calculated by multiplying the staining intensity grading score with the proportion score as previously described (36). Calculation of H-score was separately performed for membrane and cytoplasmic staining. An H-score ≤ 1 was rated as low (weak staining), whereas an H-score >1 was rated as high (strong staining) for both membrane and cytoplasm, according to the median value of the staining intensity for CXCR4 and CXCR7 as previously described (30, 37). Results of the individually assessed H-scores for the TMA cores were averaged to obtain the whole-section score for each tumor sample. In case of divergent results, slides were re-evaluated by all investigators, forming the final score by consensus.

### Statistical Analysis

Quantitative values were expressed as mean ± standard deviation or median and range as appropriate. Fisher’s exact or chi-square tests were used to analyze dichotomic variables, whereas continuous variables were investigated with a two-sided t test or Mann-Whitney Test. *P*-values <0.05 were considered statistically significant. Correlations between the staining patterns of both chemokine receptors and the clinical and histopathological data as well as among each other were calculated by Pearson and Spearman’s correlation test.

Kaplan Meier survival analysis was performed to investigate the correlation between each chemokine receptor and prognosis. Progression-free survival (PFS) was defined as the time from the date of first surgery to the first radiological evidence of disease progression or death from ACC. Overall survival (OS) was defined as the time from the date of first diagnosis to the time of death or last follow-up. Differences between survival curves were assessed by the log-rank (Mantel-Cox) test and the factors considered to independently influence survival were analyzed by Cox proportional hazard regression.

All statistical tests were performed using SPSS Statistics Version 23 (IBM) and GraphPad Prism version 8.4.1 (GraphPad, La Jolla, CA).

## Results

### mRNA Expression of Chemokine Receptors in Adrenal Tissues and NCI-H295 Cells

**Figure 1:**
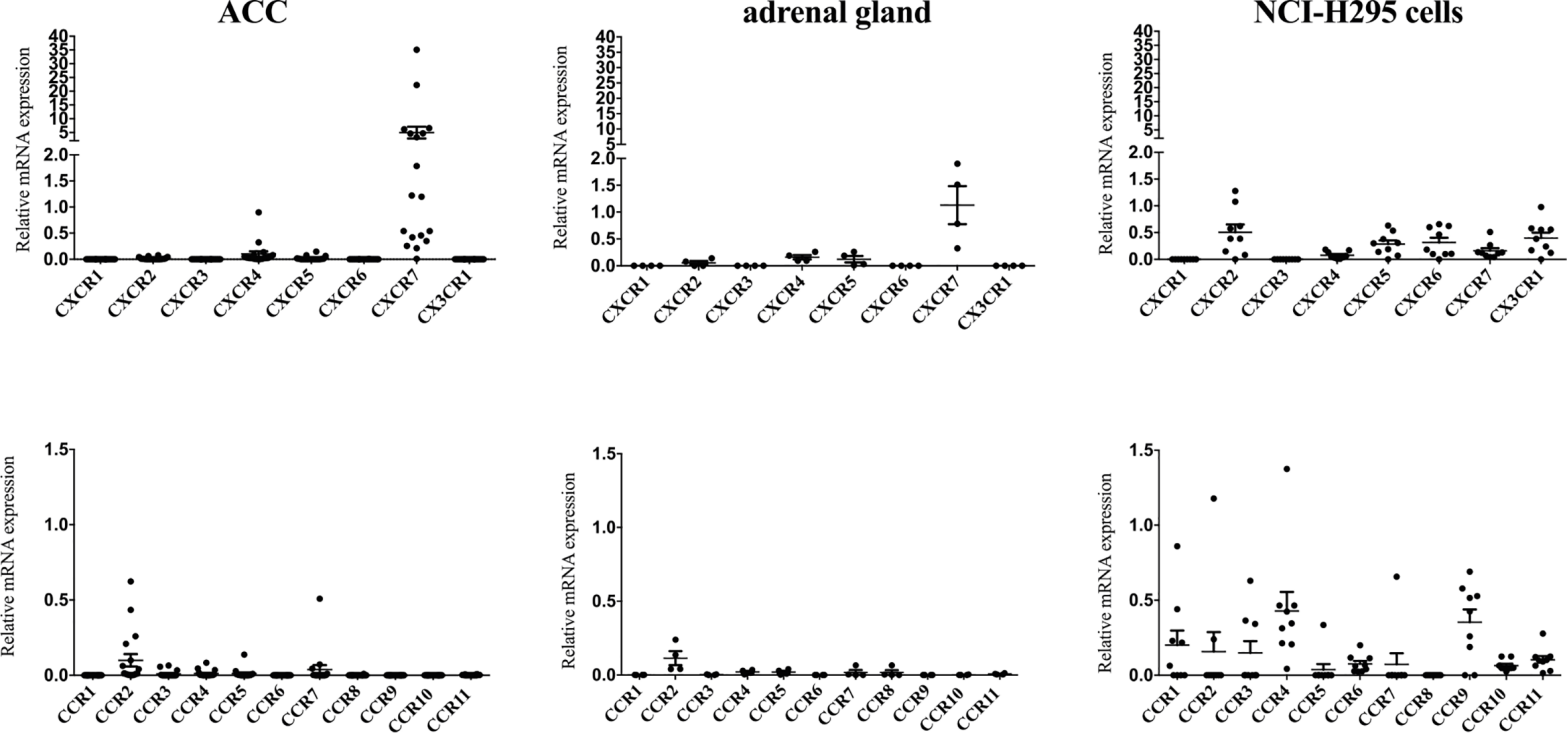
Quantitative analysis of chemokine receptor mRNA levels in adrenal tissues and NCI-H295R cells. mRNA levels of chemokine receptors were assessed by real time PCR in 18 adrenocortical carcinomas, 4 normal adrenal glands and the human adrenocortical carcinoma cell line NCI-H295R. Levels were normalized to *β-actin*. Data are given as mean ± SEM.

Relative mRNA expression of different CCR- and CXC-chemokine receptors in the normal adrenal glands (n=4) and in adrenocortical carcinomas (n=18) is reported in **Figure 1**. Each point represents the result obtained from the qRT-PCR analysis performed for a single cell sample. The highest mRNA expression levels in all analyzed samples were found for CXCR4 and CXCR7. Normal adrenals exhibited significantly higher mRNA levels for CXCR4 compared to ACCs (mean mRNA expression 1.5-fold higher in normal adrenals, *p<*0.01). CXCR4 mRNA levels in NCI-H295 were similar to those found in ACC (*p=*0.06) and normal adrenals (*p=*0.13), whereas CXCR7 mRNA levels in NCI-H295 were significantly lower compared to both ACC (46-fold lower, *p<*0.01) and normal adrenals (11-fold lower, *p<*0.01).

### Immunohistochemical Analysis of Protein Expression of CXCR4 and CXCR7 in ACC

**Figure 2:**
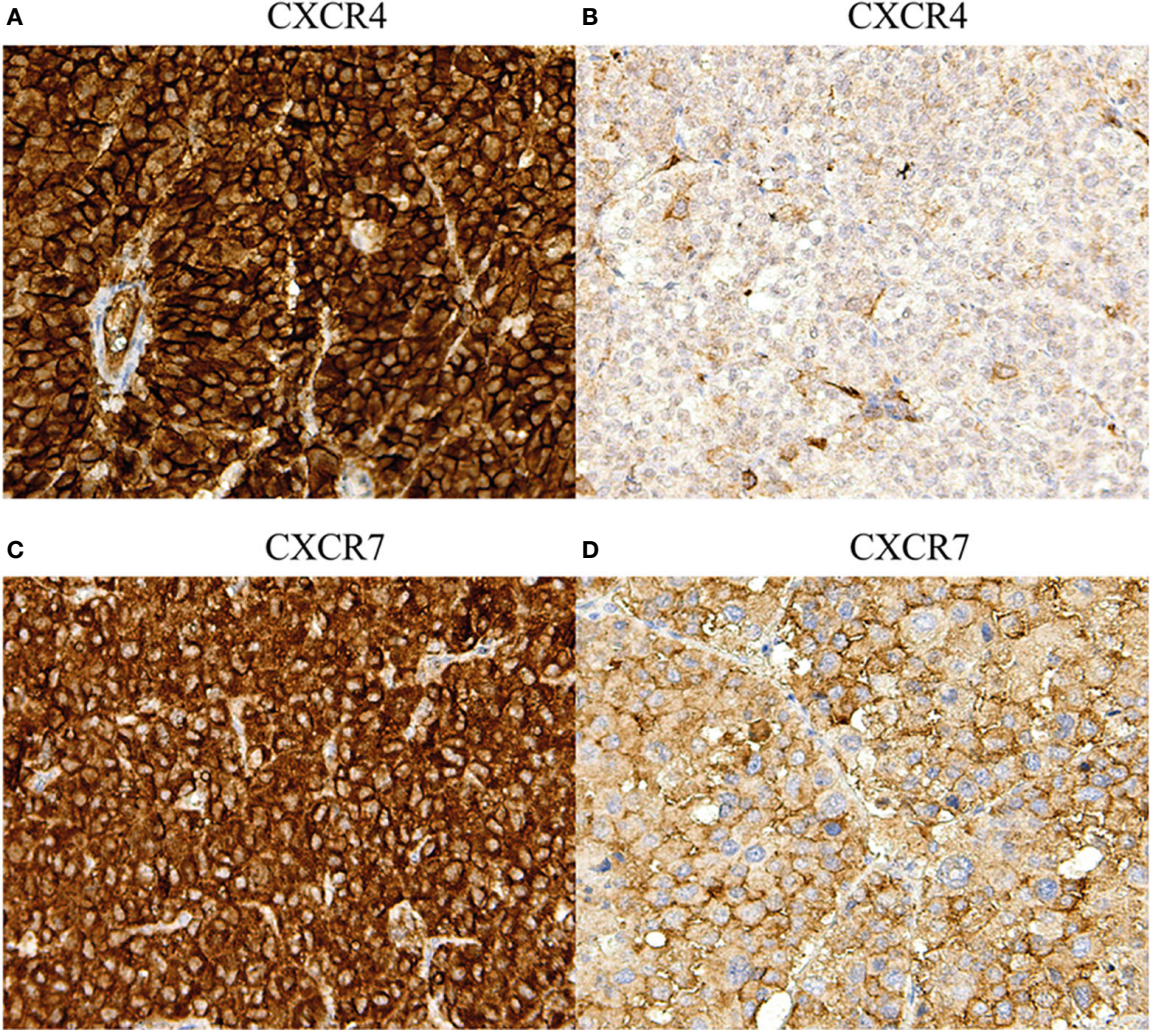
Immunohistochemical staining of CXCR4 and CXCR7 in adrenocortical carcinoma: different staining patterns. **(A)** Primary tumor, strong membranous and cytoplasmic CXCR4 staining (magnification 20x), **(B)** primary tumor, weak membranous and cytoplasmic CXCR4 staining (magnification 20x), **(C)** primary tumor, strong membranous and cytoplasmic CXCR7 staining (magnification 20x), **(D)** primary tumor, weak membranous and cytoplasmic CXCR7 staining (magnification 20x).

CXCR4 was detectable in 98% (184/187) and CXCR7 in 100% of cases (84/84). Localization at the cell membrane was preponderant for both chemokine receptors (**Figure 2**). Strong membrane staining (H-score >1) was observed in 50% of specimens both for CXCR4 (94 out 187) and CXCR7 (42 out 84 sections) (**Table 2**). Membranous and cytoplasmic staining significantly correlated for CXCR4 (r_s_=0.45, *p<*0.01) but not for CXCR7 (r=0.07, *p=*0.5). A weak correlation between the two chemokine receptors could only be seen at the cytoplasmic level (r_s_=0.32, *p<*0.01). The proportion of samples with strong CXCR4 cytoplasmic staining was higher in metastases compared to primary tumors and local recurrences (*p=*0.01 and *p=*0.04) (**Table 2**).

**Table 2:** Distribution of strong and weak cytoplasmic staining of CXCR4 among primary tumors (PT), local recurrences (LR) and metastases (M).

CXCR4	PT(n=159)	LR(n=17)	M(n=11)	p		
				PT vs M	LR vs M	PT vs LR
Cytoplasmic staining						
Strong	34 (21%)	3 (18%)	6 (55%)	0.01	0.01	ns
Weak	125 (79%)	14 (82%)	5 (45%)			
H-score (mean±SD)	1.0±0.8	0.9±0.5	1.6±1.3	ns	ns	ns
Membrane staining						
Strong	80 (50%)	9 (53%)	5 (46%)	ns	ns	ns
Weak	79 (50%)	8 (43%)	6 (54%)			
H-score (mean±SD)	1.4±1.0	1.1±0.9	1.1±1.2	ns	ns	ns

### Correlation of CXCR4 and CXCR7 Staining Intensity and Staining Pattern With Clinicopathological Features and Clinical Outcome Data in ACC

Clinicopathological features of the 187 ACC patients with complete survival data are summarized in **Table 1**. Among the 94 patients who had an initial R0 resection status, 69 (73%) developed metastases at follow-up. ENSAT tumor stage did not correlate with the staining intensity of CXCR4 or CXCR7. At membrane level, we found a weak positive correlation between CXCR4 and Ki67 (r_s_=0.17, *p=*0.028). In contrast, a weak negative correlation between CXCR7 and Ki67 was noted (r_s_=−0.254, *p=*0.03), whereas membranous CXCR7 staining was positively correlated with tumor size (r=0.3, *p=*0.02).

**Figure 3:**
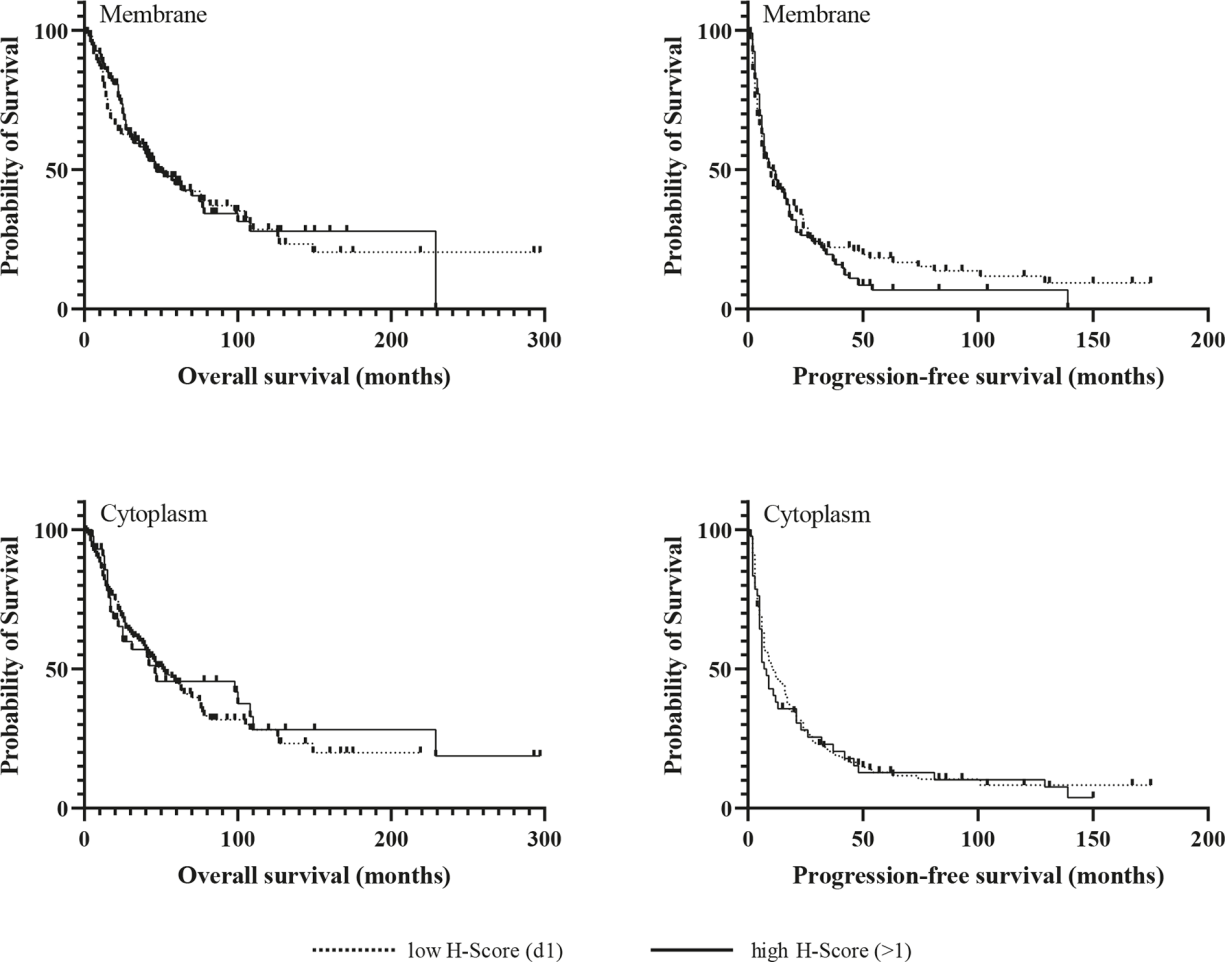
Kaplan-Meir survival analysis for overall and progression-free survival according to membranous and cytoplasmic CXCR4 expression.

**Figure 4:**
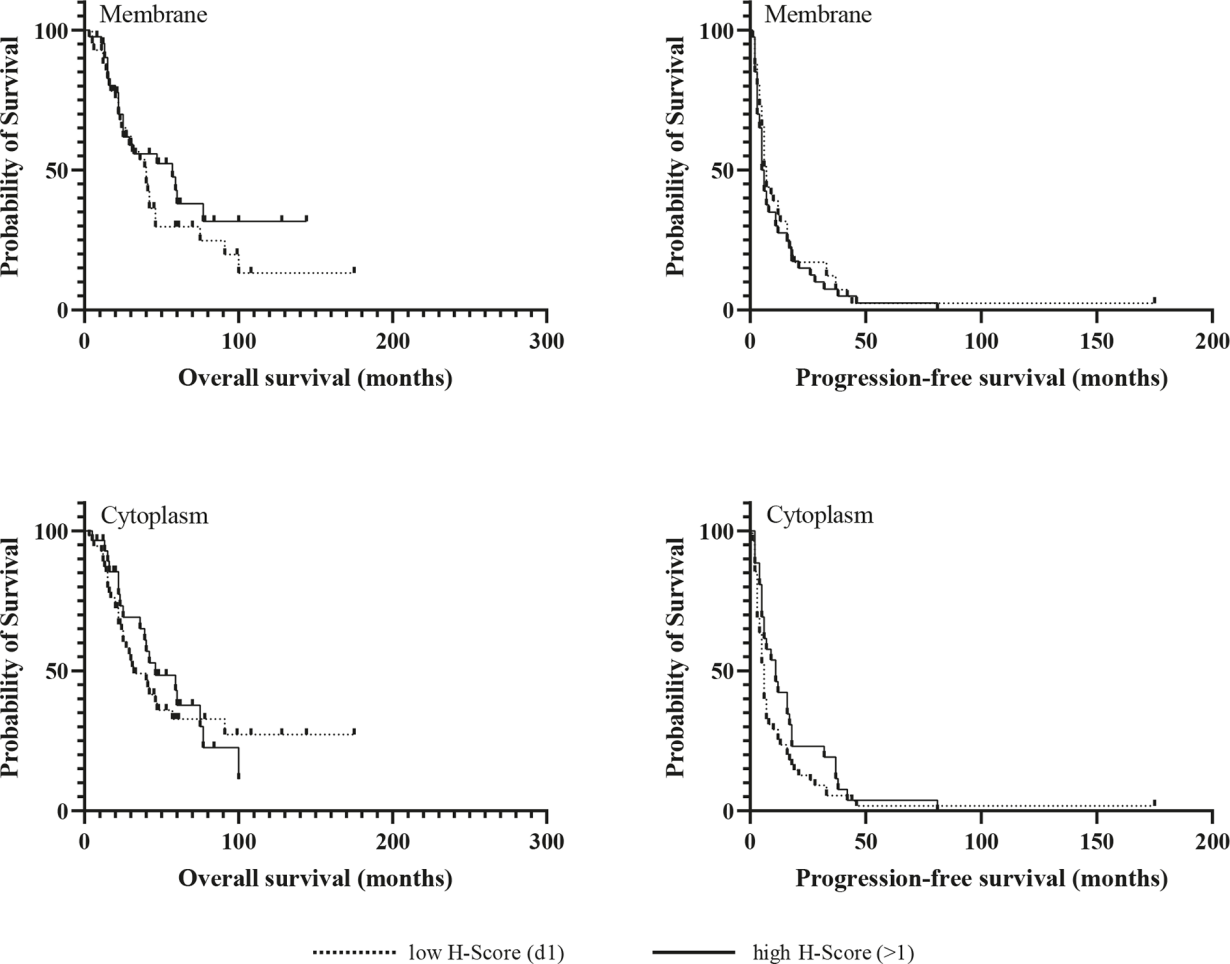
Kaplan-Meir survival analysis for overall and progression-free survival according to membranous and cytoplasmic CXCR7 expression.

Kaplan-Meier analyzes for OS and PFS revealed no significant differences between patients with high and low expression of the chemokine receptors regardless of their cellular localization, neither in the whole cohort (**Figures 3** and **4**), nor in the subgroup of patients with initial R0 resection (data not shown). In the subgroup of patients with markers of more favourable prognosis (ENSAT stage I–II, n=94; Ki67 <10%, n=38), mean PFS was significantly longer in cases with strong CXCR7 cytoplasmic staining compared to cases with weak CXCR7 cytoplasmic staining (25±21 *vs.* 12±11 months, *p=*0.04, for ENSAT I-II, and 34±4 *vs.* 8±6 months, *p=*0.02, for Ki67<10%). However, multivariate analysis did not confirm the significant association between the cytoplasmic CXCR7 staining and PFS seen in the univariate analysis in this subgroup (**Table 3**).

**Table 3:** Relationship between the immunhistochemical expression of CXCR4 and CXCR7 and progression-free survival in the subgroup of patients diagnosed at an early ENSAT stage (I–II), univariate and multivariate analysis for risk of death.

	Univariate		Multivariate	
	HR(95% CI)	p	HR(95% CI)	p
CXCR4 membrane expression				
weak (≤ 1)				
strong (>1)	0.8 (0.5–1.3)	ns		
CXCR4 cytoplasmic expression				
weak (≤ 1)				
strong (>1)	1.1 (0.7–1.9)	ns		
CXCR7 membrane expression				
weak (≤ 1)				
strong (>1)	1.1 (0.6–1.9)	ns		
CXCR7 cytoplasmic expression			0.5 (0.2–1.1)	0.1
weak (≤ 1)				
strong (>1)	2 (1.4–4.0)	0.04		
Ki67			1.2 (0.4–3.1)	0.7
low (<10%)				
high (≥10%)	1.8 (1.1–2.9)	0.02		
Weiss Score^a^			1.5 (0.7–3.3)	0.2
low (≤6)				
high (>6)	2.1 (1.1–3.9)	<0.01		
Tumor size^a^				
low (<12 cm)				
high (≥12 cm)	1.4 (0.9–2.3)	ns		
Resection status				
R0				
R1/2/x	1.1 (0.6–2.1)	ns		
Sex				
male				
female	1.0 (0.6–1.7)	ns		
Age	0.9 (0.9–1.0)	ns		
Cortisol secretion				
no				
yes	0.8 (0.5–1.3)	ns		

a Divided into low and high according to mean.

HR, hazard ratio; 95% CI, 95% confidence interval.

## Discussion

To our knowledge, this is the largest study that describes the immunohistochemical expression pattern of both chemokine receptors CXCR4 and CXCR7 in ACC and the first one that analyzes their correlation with clinicopathological parameters and clinical outcome.

We demonstrate that both chemokine receptors are highly expressed in most ACCs especially at membrane level. However, different from observations made in other malignancies, CXCR4 nor CXCR7 expression was neither associated with the occurrence of metastases nor with survival. Since both chemokine receptors are present at high levels also in normal adrenocortical tissue, it can be assumed that CXCR4 and CXCR7 are constitutively expressed by the adrenal gland and are mainly relevant for maintaining adrenal homeostasis. A recent publication from our group revealed particularly strong CXCR4 expression in the subcapsular region of the normal adrenal cortex (38), which also hosts the adrenocortical stem/progenitor cell niche (39, 40), whereas CXCR7 was uniformly distributed within all adrenocortical zones (38). A possible role for CXCR4 in the differentiation and zonation of the adrenal cortex along complimentary CXCL12 gradients was assumed (38). CXCR7, on the other hand, might be involved in less zone-specific processes such as angiogenesis or tissue repair.

Nevertheless, CXCR4 and CXCR7 might still be of therapeutic interest for ACC in the light of upcoming theranostic concepts. Especially the predominant membrane localization is of advantage as it allows direct inactivation of the chemokine receptors by ligand binding. For CXCR4, suitable radioligands are available and have been successfully tested in lymphoproliferative malignancies (19–21). Two of these radioligands, ^64^Cu-plerixafor and ^68^Ga-Pentixafor, can also reliably assess the expression of CXCR4 *in vivo* in patients affected by ACC or aldosterone producing adenoma, respectively (4, 38). Bluemel et al. went a step further towards a potential theranostic use of CXCR4 and compared the performance of ^68^Ga-pentixafor PET/CT with ^18^F-FDG PET/CT in 30 patients with advanced ACC (3). Overall, at least two thirds of the patients were rated as suitable or potentially suitable for a CXCR4-directed endoradiotherapy based on the number of lesions identified by ^68^Ga-pentixafor PET and the intensity of the tracer uptake (3).

The correlation between CXCR4 and Ki67 at membrane level suggests that the activated form of the chemokine receptor is preferentially upregulated in highly proliferative ACCs, that are known to have a dismal prognosis even after complete resection (41). Effectively blocking CXCR4 might therefore interfere with tumor growth and metastasis in ACC *in vivo*, as also highlighted by the inhibitory effect of the CXCR4 antagonist AMD3100 on the proliferation and migration of the human ACC cell line NCI-H295 reported by Kitawaki et al. (42).

The response to immunotherapy in ACC might also benefit from antagonizing CXCR4, as shown for hepatocellular carcinoma (43), pancreatic (44), breast (45) and ovarian cancer (46). These tumors escape immunosurveillance and respond poorly to immune checkpoint inhibitors due to their immunosuppressive milieu. One of the common mediators of cancer immunoresistance is the CXCL12/CXCR4 pathway due to enhanced recruitment of immunosuppressive cells in the tumor microenvironment (47). Combined blockade of CXCR4 and PD-1/PD-L1 increases antitumor immunity and significantly improves the response to immune checkpoint inhibitors (47). According to a recent analysis of the immune landscape in cancer, ACC also belongs to the leukocyte depleted tumors (“immunologically quiet”) (48). Therefore, the modest tumor response to PD-1/PD-L1 directed therapy, with a best median overall-survival of 24.9 months, is not surprising (49–51). However, recently published data from our group identified a subset of ACCs with preserved cytotoxic T-cell infiltration and significantly improved overall survival, especially in the absence of glucocorticoid excess (52). Therefore, activating tumor immunity in leukocyte depleted ACCs could be strategical in improving the response to immune checkpoint inhibitors. Several mechanisms related to leukocyte depletion and immunoresistance in ACC, such as glucocorticoid excess, upregulation of WNT/β-catenin pathway or TP53 mutations, are also associated with upregulation of CXCR4 (49–51). Glucocorticoid excess, even if not clinically manifest, is approached in the majority of cases by adrenolytic therapy with mitotane, but pharmacological targeting of WNT/β-catenin and TP53 pathways is not yet available (49, 50). Therefore, CXCR4-targeted therapies might overcome immunoresistance in ACC by simultaneously blocking multiple pathways.

As the CXCR4-specific tracer CPCR4 can be labeled with Lutetium-177 (53) and Yttrium-90 (54), endoradiotherapy of ACC may emerge as a future treatment option for patients with ACC. However, this approach requires harvesting stem cells prior to treatment initiation due to hematologic toxicity (55). This could be compromised in patients pretreated with several myelotoxic chemotherapy regimens, as is often the case with ACC.

Opposite to CXCR4, the intensity of CXCR7 membrane staining was inversely correlated with Ki67 but positively correlated with tumor size, describing thus a rather slow-growing local tumor pattern, as also reported for CXCR7-positive breast cancer samples (56). So far, one radiolabeled highly selective antibody (ACKR3-mAb) has been tested for *in vivo* assessment of CXCR7 in mice xenografted with human cancer cells showing correlation of tracer uptake with CXCR7 immunoreactivity (57).

Our study has several strengths and limitations. We assessed the immunohistochemical expression of both CXCR4 and CXCR7 in a large series of ACC samples. We used a well validated antibody shown to identify membranous and cytoplasmic CXCR4 staining both in healthy and in malignant tissues (58). However, the same antibody failed to detect CXCR4 in 25% of the analyzed ACC metastases in the study performed by Weiss et al., despite detectable CXCR4 mRNA in all samples (4). Similarly, we cannot exclude that some samples might have been classified as false-negative. We also could not investigate an equal number of tumor samples for both CXCR4 and CXCR7 and only had access to a limited pool of metastases and local recurrences unrelated to the primary tumors. Extended analyzes of the expression of both chemokine receptors and their common ligand CXCL12 not only in primary tumors but also in matched metastases together with functional studies on ACC cell lines are warranted to receive a better insight into the impact of CXCR4 and CXCR7 on the prognosis of ACC.

In summary, we could demonstrate that CXCR4 and CXCR7 are the most abundant chemokine receptors in adrenocortical carcinoma. The lack of prognostic significance and their high expression in the normal adrenal gland rather suggest a predominant role of both chemokine receptors in adrenocortical homeostasis. Nevertheless, our study provides further evidence for the theranostic potential of CXCR4 and CXCR7 in ACC, with special emphasis on potentially improving tumor response to systemic therapies.

## Data Availability Statement

The original contributions presented in the study are included in the article/supplementary materials. Further inquiries can be directed to the corresponding author.

## Ethics Statement

The studies involving human participants were reviewed and approved by Ethics committee of the medical faculty of the Julius-Maximilians University Würzburg. The patients/participants provided their written informed consent to participate in this study.

## Author Contributions

BH and SH wrote the study protocol, were involved in applications for authorities, and supervised the conduct of the study. IC did the literature research, performed the immunohistochemical analysis, statistical analysis, and data interpretation. CF performed the qRT-PCR analysis and contributed to the immunohistochemical analysis. SK provided materials for the immunohistochemical analysis. BH, BA, CR, and KL contributed to the statistical analysis and data interpretation. IC, BH, SH, MK, MF, AS, and SK co-wrote and edited the manuscript. All authors contributed to the article and approved the submitted version.

## Funding

This work was supported by the Deutsche Forschungsgemeinschaft (DFG) (within the CRC/Transregio 205/1 “The Adrenal: Central Relay in Health and Disease”), IZKF Würzburg (Grant No. F 365 to AS and SH), Deutsche Forschungsgemeinschaft (DFG AL 203/11-1 to AS and SH), and the Else Kröner-Fresenius Stiftung (Grant No. 2010_EKES.29 to SH).

## Conflict of Interest

The authors declare that the research was conducted in the absence of any commercial or financial relationships that could be construed as a potential conflict of interest.

## Abbreviations

ACC, adrenocortical carcinoma; IHC, immunohistochemistry; qRT-PCR, quantitative real-time polymerase chain reaction; RT, room temperature; FFPE, formalin fixed paraffin-embedded; ENSAT, European Network for the Study of Adrenal Tumors; OS, overall survival; PFS, progression-free survival; PET/CT, Positron Emission Tomography/Computed Tomography.
